# Effect of Engaging Trainees by Assessing Peer Performance: A Randomised Controlled Trial Using Simulated Patient Scenarios

**DOI:** 10.1155/2014/610591

**Published:** 2014-05-20

**Authors:** Charlotte Loumann Krogh, Charlotte Ringsted, Charles B. Kromann, Maria Birkvad Rasmussen, Tobias Todsen, Rasmus Lundhus Jørgensen, Rikke Borre Jacobsen, Jørgen B. Dahl, Lars Konge

**Affiliations:** ^1^Centre for Clinical Education (CEKU), University of Copenhagen and Capital Region of Denmark, Rigshospitalet, Teilumbygningen, Blegdamsvej 9, 2100 Copenhagen, Denmark; ^2^Department of Anesthesia and The Wilson Centre, 200 Elizabeth Street, 1ES-565, Toronto, Ontario, Canada M5G 2C4; ^3^Department of Anaesthesiology, Centre of Head and Orthopaedics, Rigshospitalet and Copenhagen University, Blegdamsvej 9, 2100 Copenhagen, Denmark

## Abstract

*Introduction*. The aim of this study was to explore the learning effect of engaging trainees by assessing peer performance during simulation-based training. *Methods*. Eighty-four final year medical students participated in the study. The intervention involved trainees assessing peer performance during training. Outcome measures were in-training performance and performance, both of which were measured two weeks after the course. Trainees' performances were videotaped and assessed by two expert raters using a checklist that included a global rating. Trainees' satisfaction with the training was also evaluated. *Results*. The intervention group obtained a significantly higher overall in-training performance score than the control group: mean checklist score 20.87 (SD 2.51) versus 19.14 (SD 2.65) *P* = 0.003 and mean global rating 3.25 SD (0.99) versus 2.95 (SD 1.09) *P* = 0.014. Postcourse performance did not show any significant difference between the two groups. Trainees who assessed peer performance were more satisfied with the training than those who did not: mean 6.36 (SD 1.00) versus 5.74 (SD 1.33) *P* = 0.025. *Conclusion*. Engaging trainees in the assessment of peer performance had an immediate effect on in-training performance, but not on the learning outcome measured two weeks later. Trainees had a positive attitude towards the training format.

## 1. Introduction

Assessing signs of critical illness is an essential skill for any doctor. While junior doctors often perform the initial assessment of acutely ill patients in hospitals [1] studies have shown that newly qualified doctors are poorly prepared to manage acutely ill patients [2, 3]. Hence, it is desirable to prepare final year medical students for the initial management of emergency situations.

Systematic assessment of critically ill patients using the simple ABCDE mnemonic is widely accepted as a clinical working tool [4]. The ABCDE acronym stands for airway, breathing, circulation, disability, and exposure/environment, describing the order in which the problems associated with acute illness should be addressed. This approach is applicable to all patients, as each step of the algorithm serves to assess signs of critical illness, regardless of the underlying diagnosis.

Undergraduate teaching of acute care is often inconsistent and lacks sufficient practice in core aspects of the ABCDE assessment of critically ill patients [5, 6]. The opportunities for medical students to develop and practise the ABCDE approach in emergency situations are limited. Therefore, simulation-based training that addresses the ABCDE approach may be a suitable alternative that enables trainees to practise high-risk, low-frequency emergency situations in a safe environment [7, 8]. However, the simulation-based small-group training approach is expensive in terms of utensils, mannequins, and instructors. Therefore, strategies to maximize the learning outcome should be considered.

While active participation of trainees may be an effective learning strategy, it is rarely possible to have the simultaneous participation of all members of a group, which leaves some of the trainees as passive observers. However, research suggests that observation, especially when combined with physical practice, can make a significant contribution to skill learning [9] even when observing “unskilled” demonstrators such as novices [10]. By observing other novices' practice, the trainees are typically engaged in reflection of their own performance rather than imitating the skill. According to Magill, a beneficial strategy could be to provide trainees with a checklist containing key aspects of the skill while observing [10]. The idea behind this strategy is that, under these circumstances, trainees gain a sense of involvement, which enhances motivation and encourages active problem solving and hence may have a positive influence on learning outcome and long-term retention.

The aim of this study was to explore the learning effect of engaging trainees by assessing of peer performance during a simulation-based course in which a critically ill patient was assessed. The trainees' performance was measured by performance outcome during training and two weeks after the course. The study also aimed to explore trainees' reactions to the training format.

## 2. Methods

This study was a randomised, experimental trial that compared trainees who were engaged in assessment of peer performance to trainees who were not.

### 2.1. Context of the Study

A four-hour, simulation-based ABCDE training session was developed as part of a three-week emergency medicine course that included faculty-led didactic teaching sessions on a variety of emergency medicine topics and was situated at the end of an undergraduate six-year medical curriculum.

### 2.2. Study Sample

A sample of eighty-six final year medical students attending the emergency medicine course at Rigshospitalet from 22 May until 13 June 2012 was invited to take part in the study. Eighty-four of the students accepted the invitation and were enrolled in the study. All of the trainees were at the same educational level and comparable in terms of advanced cardiac life support (ACLS) competence. A fee of 400 DKK (approximately 50 Euros) was offered for completing the study. A member of the research group randomly allocated the trainees to either the intervention or the control group using the trainees' participant ID number and randomisation sequences as well as tables created using http://www.random.org/. In both groups, the sequence of roles within the team was also part of the randomisation process.

### 2.3. Ethics

The study protocol was submitted to the Danish Bioethics Committee for the Capital Region, Copenhagen, Denmark, which waived the requirement for full ethical approval (protocol number: H-4-2012-060). Participants were informed about the purpose of the study and ensured anonymity, and individual written consents were collected.

### 2.4. ABCDE Training

The ABCDE training, including an ABCDE template (Figure 2), was designed by the research group comprising anaesthesiologists and faculty from the Centre for Clinical Education (CEKU). The ABCDE template was introduced during the first session of the emergency medicine course.The simulation-based ABCDE training session comprised an introduction by the facilitator, a video demonstration of the application of the ABCDE principles, and subsequent training on six detailed, megacode scenarios, each of which had an intended duration of 12 minutes. The cases addressed both medical and surgical conditions frequently seen in emergency departments but did not include any trauma cases. The simulation sessions were conducted in groups of six trainees on a Resusci-Anne mannequin (Laerdal Medical Corporation, Stavanger, Norway). Each group performed six scenarios facilitated by the same faculty member from CEKU. Each scenario had three active roles—a team leader and two helpers—while the rest of the group were observers. The six trainees took turns assuming these roles. All scenarios were videotaped for subsequent assessment. While facilitating the scenarios, the facilitator assessed the performances of all team leaders using a checklist scoring form (Table 2). The content of the checklist was very similar to the ABCDE template provided to all trainees at the first session of the emergency medicine course. The design of the checklist scoring form was inspired by the advanced life support Cardiac Arrest Scenario (CAS) test checklist. In addition to the checklist items, the scoring form included an overall global rating (scale 1–5), where a score ≥ 3 indicated acceptable overall performance.

After each scenario, the facilitator provided postsimulation debriefing, supplemented by comments from the peer observers; however, detailed results of the assessment were not provided.

### 2.5. Intervention Conditions

The intervention group underwent the same ABCDE training as the control group. However, peer observers of the intervention group were asked to assess the team leader's performance during each scenario using the same checklist scoring form as the facilitator. At the end of each scenario, the scoring forms of the peer observers were collected. The team leader was not informed of his/her performance score.

### 2.6. Evaluation and Retention Test

**Figure 1:**
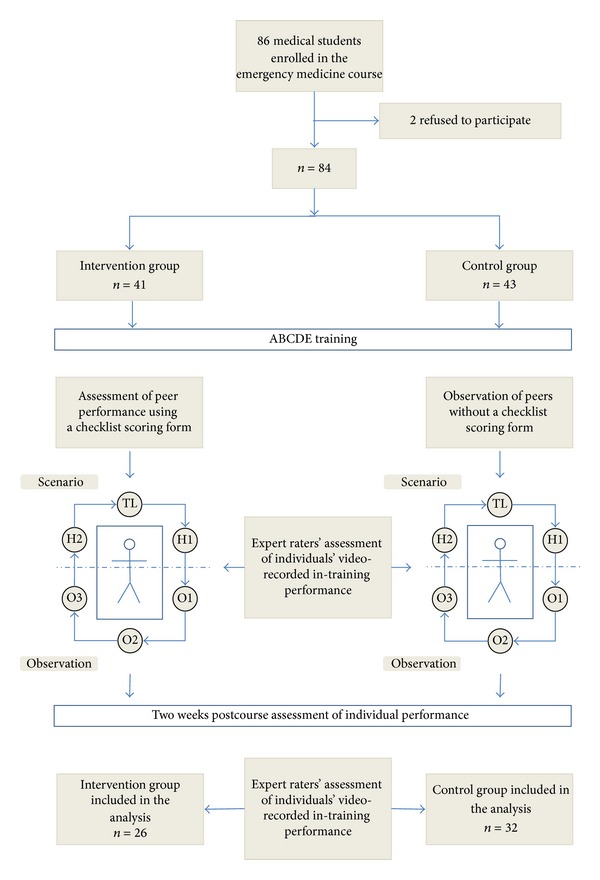
Flow chart showing the study design. Eighty-fourfinal year medical students were placed randomly into either the intervention group or the control group. Observers of the intervention group assessed the team leader's performance using a checklist scoring form. All participants were invited to participate in the assessment of performance two weeks after the course. Trainees' performances, in-training as well as after the course, were videotaped and assessed by two expert raters.

**Figure 2:**
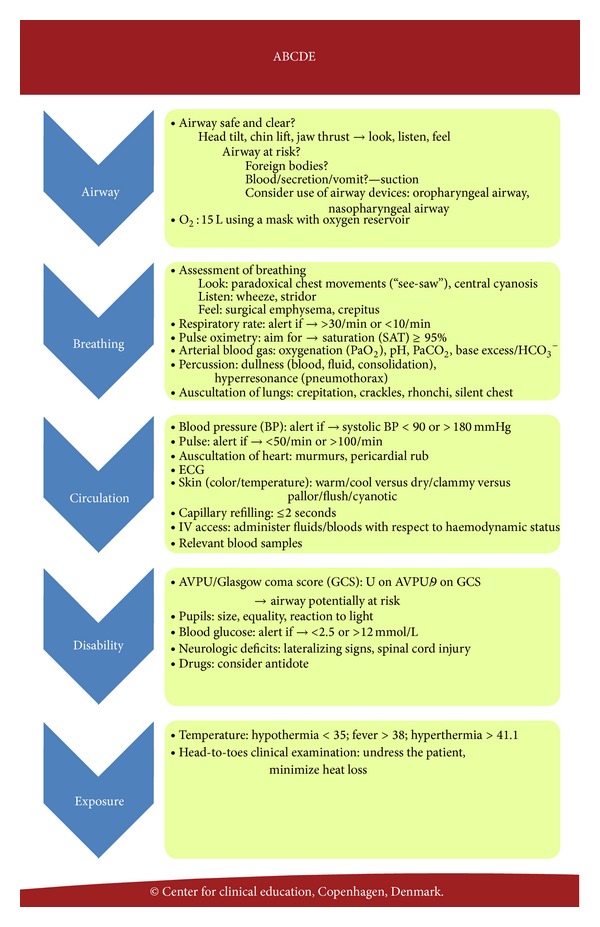
The ABCDE template describing the order in which the problems associated with acute illness should be addressed.

After the ABCDE training, the trainees answered a single evaluation question about their satisfaction with the training format; this was measured using a seven-point Likert scale. Finally, two weeks after the course, all trainees were invited to participate in the assessment of their performance. During these two weeks, the trainees did not have any further clinical or theoretical training, as the course was carried out at the conclusion of their time at the medical school. The trainees completed the performance test individually, assuming the role as team leader, and the facilitators acted as helpers. The performance test scenario was different in content but similar to the structure of the ABCDE training scenarios. As with the ABCDE training scenarios, the performance test scenario was videotaped for subsequent assessment. The study design is illustrated in Figure 1.

### 2.7. Outcome Measurements

This study included two outcome measures: the trainees' in-training performance and the trainees' individual performance assessed two weeks after the ABCDE training (retention test).

Assessments of performances were based on the video recordings obtained during training and at the retention test. For this purpose, all the videos were edited to show only the simulation sessions; the debriefings, introduction to the scenarios, and the like were edited out.

The video-recorded performances of the team leaders were assessed by two trained independent and trained raters with experience in advanced life cardiac support (ALCS) teaching and testing. The raters were blinded with regard to whether they were assessing the intervention group or the control group. The raters used the same checklist scoring form as that used by the facilitator and peer observers of the intervention group during the ABCDE training.

### 2.8. Statistical Analysis

Interrater reliability was examined using the intraclass-correlation coefficient. An average of the raters' scorings was used to compare the intervention and control groups. An independent sample* t*-test was used to compare the groups regarding overall in-training performance and performance on the retention test. The two groups were also compared regarding checklist scores, global ratings, and satisfaction with the training format. Data were analysed using the PASW statistical software package version 19.0 (SPSS Inc., Chicago, Illinois, USA). *P* values < 0.05 were considered statistically significant.

## 3. Results

Eighty-four out of the 86 trainees agreed to participate and all 84 completed the ABCDE training.

Interrater reliability was high on both the checklist score and the global rating (ICC = 0.83 and 0.79, resp.).

The intervention group obtained a significantly higher overall in-training performance score than the control group: mean checklist score of 20.87 (SD 2.51) versus 19.14 (SD 2.65) *P* = 0.003 and mean global rating of 3.25 SD (0.99) versus 2.95 (SD 1.09) *P* = 0.014.

Students' evaluation of the training format was significantly higher in the intervention group: mean rating of 6.36 (SD 1.00) versus 5.74 (SD 1.33) in the control group, *P* = 0.025.

The dropout rate at the retention test was rather high in both groups: 15 participants (37 percent) in the intervention group and 11 participants (26 percent) in the control group, *P* = 0.64.

The learning outcome, assessed two weeks after the course, showed no significant difference between the two groups, regarding either checklist scores (*P* = 0.923) or global ratings (*P* = 0.322) (Table 1).

## 4. Discussion

This study has demonstrated that engaging trainees in structured assessment of their peers during observational phases in a simulation-based ABCDE training session had a positive effect on in-training performance. Through a simultaneous combination of observation and assessment of peer performance, the trainees of the intervention group were offered the opportunity to extract features of the performance in order to guide and develop their own performance of the skill [10]. However, the results of the retention test demonstrated no significant difference between the two groups.

Students' attitudes towards assessment of peer performance highly endorsed the concept of active observation during ABCDE training. Assessment of peer performance has been increasingly adopted at a number of levels in the education of healthcare professionals [11, 12]. Introducing assessment of peer performance in undergraduate medical education may offer insights into the students' work habits and those of their peers, which could foster motivation and reflection of personal and professional competences. Additional advantages of assessment of peer performance include familiarisation with peer review from colleagues and promotion of future learning and professional development [13].

A phenomenon known as test-enhanced learning could be relevant to this study. Test-enhanced learning implies that being tested will enhance the long-term retention of knowledge or skills. Hence, being tested in itself should affect long-term retention positively [14, 15]. Kromann et al. investigated testing as part of a simulation-based resuscitation course and found a significant higher learning outcome in the intervention group, indicating that testing, in itself, may be an effective strategy to increase learning outcome [15].

Observation, combined with simultaneous assessment of peer performance, could generate a sense of a “testing effect.” However, in our experimental setup both the intervention group and the control group were tested during the training (e.g., the facilitator used the checklist scoring form during scenario training in both groups, video recordings of all scenarios, etc.). This meant that we were not able to investigate the effect of testing.

Using e-learning programs could be a feasible strategy to prolong retention after simulation-based skill courses. However, Jensen et al. found that e-learning had no significant effect as a booster to maintain competences following an advanced life support (ALS) course. The lack of social interaction was identified as the major cause predicting the use of e-learning [16]. Future studies that combine tests with e-learning could demonstrate prolonged retention of skills obtained in a simulated setting.

DeMaria et al. found that participants exposed to emotional stress demonstrated greater anxiety, which correlated with increased ACLS scores measured six months after the course [17]. The participants of our study were exposed to continuous assessment and video recording, which could have generated emotional stress; however, no objective measurement of this aspect was conducted.

One strength of this study is measuring learning outcomes two weeks after the course (retention test). Potential improvements in learning measured by later tests are in accordance with general recommendations for evaluation of skill learning, that is, to test learning outcomes after a pause in training (retention of learning) in order to prove sustainable skills [18]. Furthermore, using two raters with experience in ALS training and assessment of skills ensured reliable ratings.

This study has certain limitations. The study includes a risk of selection bias, as it is usually the most competent students who volunteer for educational studies. Having said that, almost 97 percent of the sample volunteered to participate. There is also a risk of selection bias due to the fee for participating in the study. However, the randomised design and the relatively large sample aimed to minimise selection bias. Furthermore, the dropout rate was almost similar in both groups, despite the reward. The high dropout rate could possibly be explained by a lack of motivation due to the fact that the trainees had just qualified as doctors and might not have felt the need for any additional training.

The students in the intervention group rated the training format higher than the control group and hence engaging students in peer assessment during ABCDE training may represent a valuable addition. However, future research is required to investigate how and if the assessment of peer performance during ABCDE training enhances knowledge and understanding and long-term learning outcome.

## 5. Conclusions

Engaging trainees in the assessment of peer performance using a checklist scoring form during observational phases in a four-hour, simulation-based ABCDE training course had an immediate effect on in-training performance but not on learning outcome measured two weeks later. In addition, trainees had a positive attitude towards assessment of peer performance.

## Figures and Tables

**Table 1:** Performance of the intervention group compared with performance of the control group measured as in-training performance and performance two weeks post-course.

	Intervention group	Control group	
In-training performance	*N* = 41	*N* = 43	*P*-value
	Mean (SD)	Mean (SD)	
Checklist scores	20.87 (SD 2.51)	19.14 (SD 2.65)	0.003
Global rating	3.52 (SD 0.99)	2.95 (SD 1.09)	0.014

Two weeks post-course performance	*N* = 26	*N* = 32	*P*-value
	Mean (SD)	Mean (SD)	

Checklist scores	19.90 (SD 2.89)	19.84 (SD 1.75)	0.923
Global rating	3.52 (SD 0.98)	3.73 (SD 0.65)	0.322

Definition of abbreviations: SD: standard deviation.

**Table 2:** The checklist scoring form. The intervention group used the checklist to assess peer performance during the observational phases. In addition, the facilitator and the two raters assessing in-training performance and performance two weeks post-course used the same checklist.

	A—Airway	Tick the box if performed
1	Assess if the airway is open (patient talks/has normal respiration)	
2	Ask helper to apply Hudson mask with reservoir and connect 10–15 L O2	

	B—Breathing	Tick the box if performed

3	Assess respiration (looks, listens, feels)	
4	Ask for respiratory frequency	
5	Ask helper to apply pulse oximetry	
6	Perform auscultation of the lungs	

	C—Circulation	Tick the box if performed

7	Ask helper to perform blood pressure measurement
8	Ask helper to assess central pulse	
9	Ask helper to measure ECG	
10	Assess the skin: color/temperature	
11	Assess capillary response	
12	Perform auscultation of the heart	
13	Ask helper to do a intravenous canulation	
14	Ask helper to do an ABG	
15	Ask helper to take out blood samples	

	D—Disability	Tick the box if performed

16	Assess if the patient is concious (AVPU/GCS)	
17	Assess and estimate size of pupiles	
18	Ask for blood sugar value.	
19	Examine motor function of limbs	

	E—Exposure/Enviroment	Tick the box if performed

20	Ask helper to measure temperature	
21	Head-to-toe examination	

	Analysis	Tick the box if performed

22	Summary of ABCDE assessment	
23	Correct analysis of ABG	

	Diagnostics and treatment	Tick the box if performed

24	Propose relevant diagnosis	
25	Outline clinical course	

	Global rating:	Scale: 1–5 (Acceptable ≥ 3)

	Overall assessment of team leader's performance	

Definition of abbreviations:

ECG: electrocardiogram.

ABG: arterial blood gas.

AVPU: Alert, Verbal, Pain and Unconsious.

GCS: Glascow Coma Scale.
